# Treat and extend regimen with aflibercept for chronic central retinal vein occlusions: 2 year results of the NEWTON study

**DOI:** 10.1186/s40942-019-0159-x

**Published:** 2019-04-15

**Authors:** Rahul N. Khurana, Louis K. Chang, Alok S. Bansal, James D. Palmer, Chengqing Wu, Mark R. Wieland

**Affiliations:** 1 Northern California Retina Vitreous Associates, 2485 Hospital Drive, Suite #200, Mountain View, CA 94040 USA; 2 Department of Ophthalmology, University of California, San Francisco, San Francisco, CA USA; 3 Celgene Corporation, Berkeley Heights, NJ USA

**Keywords:** Central retinal vein occlusion, Macular edema, Chronic, Treat and extend, Aflibercept, Ranibizumab, Bevacizumab, Non-ischemic

## Abstract

**Background:**

To determine whether aflibercept (Eylea; Regeneron Pharmaceuticals, Inc., Tarrytown, NY) could continue to extend the macular edema free interval in patients on a treat and extend (TAE) with non-ischemic central retinal vein occlusions (CRVOs) previously treated with ranibizumab (Lucentis; Genentech, Inc., South San Francisco, CA) or bevacizumab (Avastin; Genentech, Inc., South San Francisco, CA) in the second year.

**Methods:**

Twenty patients with macular edema secondary to non-ischemic CRVOs previously treated with ranibizumab or bevacizumab were prospectively treated with intravitreal aflibercept injection (IAI) using a TAE dosing regimen. Injection frequencies were extended 2 weeks if there were no signs of disease activity on OCT or change in visual acuity. In the second year of the study, patients who have recurrences of macular edema could be re-challenged with a longer treatment interval under the following criterion: absence of any macular edema on three consecutive visits with the same treatment interval.

**Results:**

Twenty patients had an average duration of a CRVO for 22 months (range 7–90) and averaged an anti-VEGF treatment every 42 days (range 28–60 days). The macular edema free interval increased from 38 to 75 days when switched to aflibercept (p = 0.000003) at month 24. There was an average increase of 37 days (median 34 days; range 0–91 days) in the macular edema free interval with aflibercept. At the month 24 visit, 50% (8/16) went > 12 weeks with a macular edema free interval between IAI. There was an improvement in vision (+ 8 ETDRS letters, p = 0.006) and decreased retinal thickness (158 µm, p = 0.00003) with aflibercept treatment at month 24.

**Conclusions:**

The 2-year results of the NEWTON study demonstrated the sustained benefits of a TAE dosing regimen with aflibercept in patients with chronic CRVOs. The visual acuity gains and anatomic improvements observed at year one were maintained through month 24 with less visits and treatments. This may help minimize the treatment burden in patients with recurrent macular edema secondary to non-ischemic CRVO.

*Trial Registration *ClinicalTrials.gov, NCT01870427, Registered June 6, 2013, https://clinicaltrials.gov/ct2/show/NCT01870427?cond=NEWTON&rank=1.

Presented at the RETICON 2017: The Retina Congress with Live Surgery, Chennai, India-April 2017.

## Background

The acute thrombosis of the central retinal vein at the level of the lamina cribosa resulting from local structural factors (i.e. atherosclerosis of the central retinal artery) or occlusion from intraocular inflammation is suspected to play a role in the pathogenesis of central retinal vein occlusion (CRVO) [1]. Occlusion of the central retinal vein results in hypoxia and upregulation of proinflammatory mediators and vascular endothelial growth factor (VEGF) [2]. Inhibition of VEGF by intravitreal medications (ranibizumab (Lucentis; Genenetch, South San Francisco, CA), bevacizumab (Avastin; Genentech, South San Francisco, CA) and aflibercept (Eylea; Regeneron Pharmaceuticals, Tarrytown, NY) and intravitreal corticosteroids are effective for the treatment of macular edema associated with CRVOs but the primary endpoint in those clinical trials was only at 6 months [3–6].

Long-term follow-up of these patients revealed that the macular edema associated with CRVO requires chronic treatment beyond 6 months [7–10]. Through 4 years of follow-up in the RETAIN study, 56% of CRVO patients still required anti-VEGF injections to treat recurrent edema (every 2 months) and in those patients the mean number of injections was 28.4. Even though the recurrent edema resolved with treatment, there was macular damage due to recurrent edema and these patients had a guarded visual prognosis [8].

Intravitreal aflibercept injection for previously treated macular edema associated with central retinal vein occlusions (NEWTON) was a clinical trial that examined whether aflibercept could extend the macular edema treatment free intervals in patients with chronic retinal vein occlusions (mean duration of 22 months). At 1 year, patients

with chronic CRVOs managed with ranibizumab or bevacizumab on a treat and extend (TAE) regimen could be successfully transitioned to aflibercept while maintaining their excellent visual and anatomical outcomes on a longer treatment interval [11]. The purpose of this paper is to report the second year results of the NEWTON clinical trial.

## Methods

The NEWTON study is a prospective, phase IV, single-center, open-label clinical trial (ClinicalTrials.gov identifier NCT01870427). Before the study was initiated, El Camino institutional review board (Mountain View, CA) approved the trial, and all patients gave written informed consent prior to enrollment. Clinical data were collected at Northern California Retina Vitreous Associates (Mountain View, CA). It was conducted in compliance with all institutional regulations concerning the ethical use of human volunteers, the Health Insurance Portability and Accountability Act, and the Declaration of Helsinki.

Study methods including inclusion criteria have been reported previously [11]. Briefly, twenty patients with macular edema secondary to chronic non-ischemic CRVOs were switched to intravitreal aflibercept injection (IAI) (2.0 mg) on a TAE dosing regimen. Non-ischemic CRVOs were defined by less than 10 disc areas of non-perfusion (retinal capillary dropout) by fluorescein angiography and the absence of any iris or retinal neovascularization. Patients must have received treatment for at least 6 months and 3 initial loading doses of anti-VEGF injections, evidence of recurrence of macular edema when extended beyond 4 weeks on a TAE regimen [11]. The extension criteria included loss of < 5 Early Treatment Diabetic Retinopathy Study (ETDRS) letters of vision since the previous visit and absence of any macular edema on OCT imaging. If both of the extension criteria were met, the patient received a treatment with IAI, and the interval to the next visit was extended by 2 weeks. If both of the extension criteria were not met on a follow-up visit, the patient received a treatment with IAI, but the interval to the next follow-up visit was further reduced by 1 week, until both the extension criteria were met or the minimum interval of 4 weeks was reached.

During the first year of the study, after decreasing the treatment interval until extension criteria were once again met, the injection interval was not lengthened even if extension criteria were met at subsequent follow-up visits. The interval was maintained for the remainder of the first year of the study. In the second year, patients who no longer had macular edema after a recurrence during the study could be re-challenged with a longer treatment interval if there was no macular edema on three consecutive visits with the same treatment interval. In these cases, the treatment interval was extended by 1 week. The interval was further increased by 1 week after any visit at which macular edema was not present/extension criteria were met. If recurrence of macular edema was noted/extension criteria not met, the interval was shortened by 1 week.

No other therapies for macular edema secondary to CRVO, such as intravitreal corticosteroids or laser photocoagulation, were allowed at any time point during the study treatment period.

The statistical significance of the mean change from baseline in macular edema free treatment interval, ETDRS BCVA, central subfoveal retinal thickness (CST) and total macular volume (TMV) at month 24 was evaluated using the Wilcoxon signed-rank test. Last observation carried forward was performed for the patients that did not complete the month 24 follow-up. A p value < 0.05 was considered statistically significant. All statistical tests were performed in Statistical Analysis System (SAS) version 9.2 (SAS Institute, Cary, NC).

## Results

Twenty patients with non-ischemic CRVOs with ETDRS BCVA between 83 and 34 letters (Snellen equivalent range 20/25–20/200) were enrolled in the study and treated with intravitreal aflibercept. The baseline characteristics of the twenty patients are listed in Table 1. The mean duration of macular edema secondary to CRVO was 22 months (range 7–90 months). Among the seventeen patients (85%) completing month 12, sixteen patients (94%) completed month 24 with 1 patient withdrawing due to relocation at week 61. In total, 16 out of 20 enrolled patients (80%) completed month 24, and of the 100 possible visits from month 12 through month 24, 2 (2%) were missed.

**Table 1:** Patient demographics and baseline characteristics

Patient number	Age	Gender	Race	Comorbidities	Duration of CRVO (months)	Previous anti-VEGF agent	# of injections	Previous CME free interval (days)	BCVA	Central retinal thickness (µm)	Total macular volume (mm^3^)
001	75	F	White	HTN	12.7	Ranibizumab	10	35	20/32	303	8.89
002	86	M	White	HTN	45.5	Bevacizumab	34	35	20/50	336	8.84
003	70	M	White	DM & HTN	13.2	Ranibizumab	10	37	20/32	458	9.3
004	38	F	Hispanic	None	10.5	Ranibizumab	8	42	20/50	269	8.05
005	73	M	White	HTN	22.1	Ranibizumab	14	45	20/32	283	7.34
006	92	F	White	HTN	17.8	Ranibizumab	9	56	20/80	485	8.67
007	82	M	White	HTN	10.9	Ranibizumab	9	28	20/32	482	10.63
008	81	M	Asian	None	15.7	Ranibizumab	11	42	20/25	282	8.98
009	85	F	Hispanic	HTN	8.5	Ranibizumab	7	42	20/200	591	11.43
010	71	F	White	None	58.1	Bevacizumab	47	35	20/40	355	7.96
011	70	M	Hispanic	HTN	89.8	Bevacizumab	37	42	20/250	736	14.1
012	87	F	Hispanic	HTN	10.2	Ranibizumab	7	42	20/160	450	9.16
013	65	F	Hispanic	None	7.7	Bevacizumab	6	35	20/25	344	9.59
014	78	F	White	HTN	7.8	Ranibizumab	6	56	20/50	302	8.49
015	61	M	White	None	8.5	Ranibizumab	6	35	20/125	512	9.75
016	81	F	Hispanic	HTN	20.0	Bevacizumab	12	42	20/25	538	8.37
017	51	M	White	HTN	6.4	Bevacizumab	6	28	20/25	355	9.16
018	68	F	White	DM & HTN	31.5	Ranibizumab	18	28	20/200	772	11.7
019	55	M	Asian	None	8.5	Ranibizumab	6	35	20/32	279	8.53
020	78	M	White	HTN	30.4	Bevacizumab	31	28	20/200	559	13.56

*VEGF* vascular endothelial growth factor, *CME* cystoid macular edema, *HTN* hypertension, *DM* diabetes mellitus

### Macula edema free interval

The primary endpoint of the study was macular edema free interval at 12 months. The macula edema free interval increased from 38 days at baseline to 64 days at month 12 (p = 0.000003) to 75 days at 24 months (p = 0.000003) [11]. The mean increase in the macular edema free interval at 24 months from baseline is 37 days (median 34 days; range 0–91 days).

**Fig. 1:**
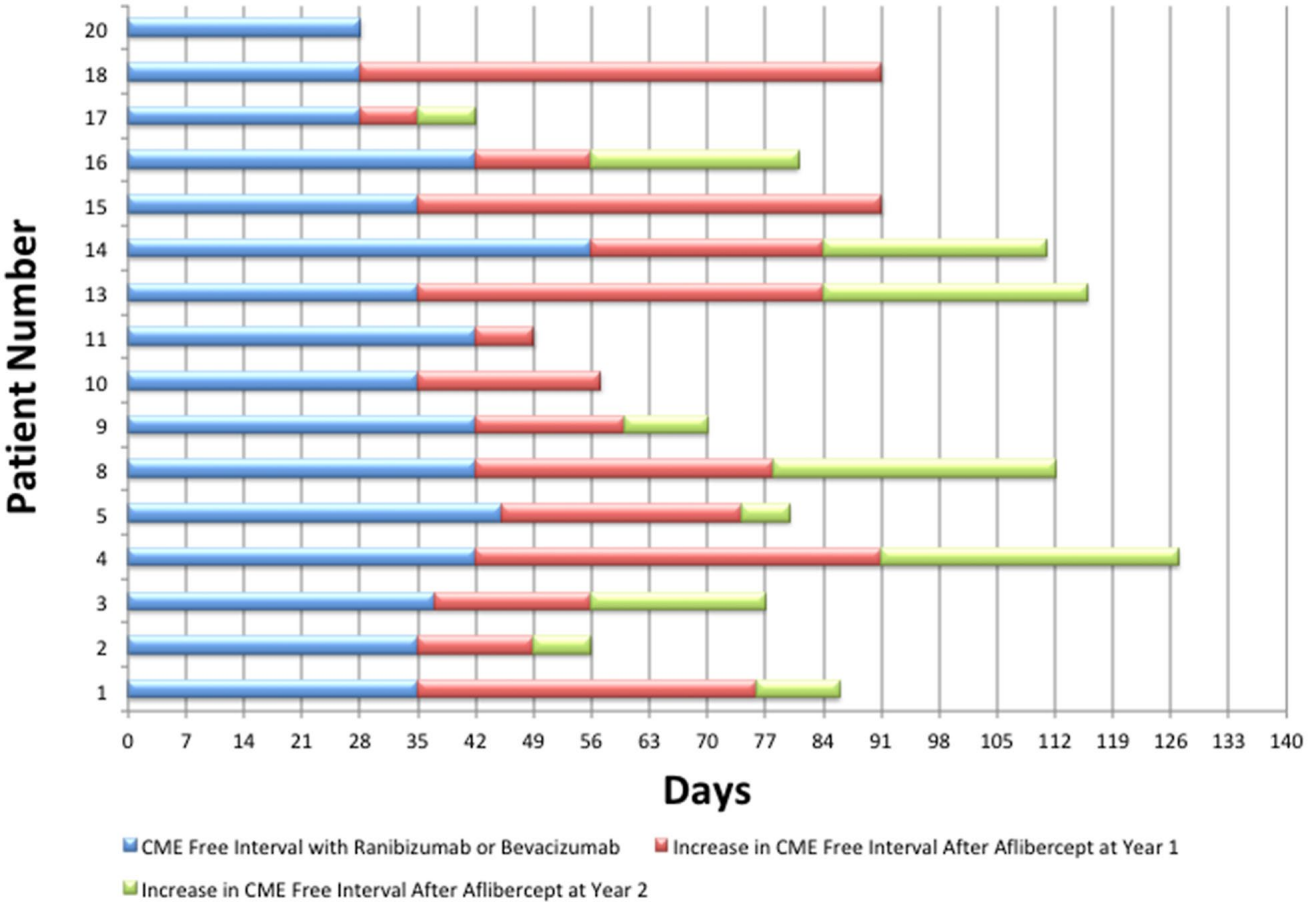
Macular edema free interval with aflibercept at year 2

At the month 24 visit, 94% of the patients (15/16) had an increase in their macular edema-free interval with IAI compared to the prior interval with either bevacizumab or ranibizumab and 69% (11/16) had an increase in their macular-free interval with IAI in year 2 compared to year 1 with IAI. These individual changes are illustrated in Fig. 1. At month 24 visit, 25% of the patients (4/16) had extended their macular edema free interval without macular edema recurrence with IAI during the entire 2 year study period. At the month 24 visit, 50% (8/16) went > 12 weeks with a macular edema free interval between IAI.

Among the 11 patients who were re-challenged in the second year, 55% (6/11) were successfully transitioned to a longer macular edema free interval from 55 days at year one to 69 days at year two. 45% (5/11) of the patients whose macular edema free interval was attempted to be lengthened were unsuccessful with recurrence of macular edema on the longer interval.

### Treatments and visits

From month 12 through month 24, the mean number of office visits and treatments ± standard deviation (SD) was 6 ± 2 (range 4–12 visits).

### Visual acuity

The BCVA gains through month 12 were maintained through month 24. The mean BCVA ± SD at baseline was 62 ± 18 letters (20/63 Snellen equivalent), at month 12 was 68 ± 20 letters (20/40 Snellen equivalent) and at month 24 was 70 ± 18 letters (20/40 Snellen equivalent). The mean BCVA improved 8 ETDRS letters with IAI by month 24 from baseline (p = 0.006).

At the month 24 visit, 75% of patients (12/16) had a BCVA of 20/40 or better [compared with 50% (8/16 patients) at baseline].

### Anatomical outcomes

Improvements in the retinal thickness achieved through month 12 were maintained through month 24. At baseline, mean CST (± SD) was 434 ± 150 μm. There was a significant decrease of 158 ± 169 μm from baseline in mean CST (p = 0.00003) to 276 ± 67 μm at month 24. At baseline, the mean TMV (± SD) was 9.6 ± 1.8 mm^3^. There was a significant decrease of 1.7 ± 2.0 mm^3^ from baseline in mean TMV (p = 0.00002) to 7.9 ± 0.9 mm^3^ at month 24. At month 24, 94% (15/16) of the patients did not have any macular edema [compared with 31% (5/16) at baseline].

### Safety

There were no ocular adverse events during the second year (There was one case of a combined macular hole and rhegmatogenous retinal detachment that developed 11 weeks after IAI during the first year). There were no cases of endophthalmitis nor ocular inflammation after the 91 intravitreal injections in the second year.

## Discussion

The 2-year results of the NEWTON study demonstrated the sustained benefits of a TAE dosing regimen with aflibercept in patients with chronic CRVOs (mean duration, 22 months). The visual acuity gains and anatomic improvements observed at year 1 were maintained through month 24. The macular edema free interval increased by approximately 5 weeks (37 days) compared to baseline further minimizing the burden of the disease in this chronic condition.

The long-term management of macular edema secondary to CRVOs is challenging as many patients require more frequent monitoring and treatments compared to branch retinal vein occlusions [8]. After initially resolving the cystoid macular edema secondary to the CRVO with fixed dosing, what is the ideal strategy moving forward to prevent recurrences and maintain the initial visual acuity gains over the long term during the maintenance phase? In the COPERNICUS Trial, treatment with fixed monthly aflibercept over 24 weeks resulted in rapid and sustained improvements in visual and anatomic end points that were subsequently maintained with as needed (pro re nata (PRN)) dosing with monthly evaluations through year 1 [4, 12]. However, in the second year of the COPERNICUS trial, a PRN dosing regimen with monitoring every three months, with treatment only following disease recurrence, did not maintain the visual and anatomic improvements achieved after a fixed monthly dosing regimen [9]. In the HORIZON-Retinal Vein Occlusion study, an extension study that included patients who completed Central Retinal Vein Occlusion study: Evaluation of Efficacy and Safety Trial (CRUISE), a PRN dosing regimen with ranibizumab with monitoring every 3 months in the second year also showed loss of the initial visual acuity gains [7]. On the other hand, the CRYSTAL study which utilized ranibizumab on a PRN dosing regimen with monthly monitoring during the first year with the option to be monitored every 2 months during the second year maintained the early visual acuity gains [13]. This suggests that a 2 month monitoring interval maybe safer than quarterly interval with PRN dosing.

An alternative management strategy to PRN in the long-term management of macular edema secondary to CRVOs is TAE. It is the most common regimen utilized by > 70% of retinal specialists in the United States in 2017 [14]. The efficacy of TAE dosing for anti-VEGF therapy was first demonstrated in the treatment of neovascular age-related macular degeneration [15–17]. By proactively treating these patients at each visit using an individualized dosing interval approach, the goal is to minimize the recurrences of CME that could lead to irreversible vision loss with PRN dosing regimens while decreasing the treatment burden with extended visit intervals as compared to monthly treatment. The Study of Comparative Treatments for Retinal Vein Occlusion 2 (SCORE2) trial compared aflibercept and bevacizumab TAE dosing with monthly dosing for the following 6 months after the initial 6 month fixed dosing period in patients who demonstrated a good response at month 6. There was an average of 1 to 2 fewer injections in TAE compared with monthly but due to the wide confidence intervals on the visual acuity differences between the two regimens, caution was advised before concluding that TAE and monthly dosing outcomes are the same [18].

The NEWTON clinical trial switched patients with chronic CRVOs (mean duration, 22 months; range 7–90 months) previously managed with ranibizumab or bevacizumab to aflibercept on a TAE regimen with a longer macular edema free interval (approximately 4 weeks) while maintaining their excellent visual and anatomical outcomes achieved at 1 year. In the second year, the mean macular free interval was extended another 11 days while preserving the visual acuity and anatomical gains in the second year. Increasing the CME free interval from 38 to 74 days corresponds to a reduction of yearly office visits from 9 to 5. Furthermore, half of the patients went > 12 weeks between visits with the TAE regimen with aflibercept. The extension of the macular free interval decreases the number of visits, diagnostic tests and treatments, which is especially important in this chronic condition.

During the second year, some patients were re-challenged to extend their CME free interval if they had been stable over the previous three intervals. Nearly half of the patients (55%) were able to go longer with IAI. However, 45% could not be further extended with IAI highlighting the need for regular monitoring and treatment. There are certain patients with chronic CRVOs whose macular edema regularly recurs even with aflibercept, further illustrating the burden of this condition over time. Due to the small numbers in this study, we are unable to predict which patients’ treatment intervals could be extended from their baseline features.

The strengths of this study are the prospective design, good protocol adherence to a TAE regimen, and a well-defined patient cohort with long-standing disease (mean duration, 22 months) managed for an additional 2 years. The limitations of the study include a relatively small sample size (n = 20) and the lack of a control group. It is important to emphasize the limited published evidence regarding the long-term outcomes of patients with CRVOs with small sample sizes. The RETAIN study included thirty two CRVO patients while another retrospective study of long term outcomes in CRVOs (4 years) in routine clinical practice included twenty five patients [19]. The SCORE2 trial will be assessing patients at year 5 and this will be helpful to further understand the long term prognosis of CRVOs. There is also a possibility that the increased treatment free interval could be associated with a natural decrease in disease severity over time (not related exclusively to a particular drug nor treatment regimen)

## Conclusions

The 2-year results of the NEWTON clinical trial affirm that patients with chronic CRVOs could maintain their excellent visual outcomes achieved with IAI on a TAE regimen through the second year. These CRVO patients were able to preserve the visual gains and anatomical improvements observed during the first year with a TAE regimen with less visits and subsequent treatments in the second year. The macular free interval increased by approximately 5 weeks (37 days) compared to their treatment interval with ranibizumab or bevacizumab further minimizing the burden of the disease following treatment with aflibercept in this chronic condition.
